# The Detection of Lyman Alpha Radiation Formed by the Slowing Down of Protons and Tritons Produced by the ^3^He (n, tp) Reaction—A Model Study

**DOI:** 10.6028/jres.114.012

**Published:** 2009-06-01

**Authors:** John W. Cooper, Michael A. Coplan, Patrick P. Hughes

**Affiliations:** Institute for Physical Science and Technology, University of Maryland, College Park, MD 20742; Institute of Physical Sciences and Technology, University of Maryland, College Park, MD 20742Ionizing Radiation Division, National Institute of Standards and Technology, Gaithersburg, MD 20899-8461

**Keywords:** charge exchange, computer modeling, Lyman alpha radiation, neutron detection

## Abstract

The observation of Lyman alpha (L*α*) radiation produced by the end products of the ^3^He (*n*,*tp*) reaction has suggested the possibility of a new method of cold thermal neutron detection. In order for this goal to be achieved, a basic understanding of how the L*α* radiation is formed and how it may be detected, is needed. The model study described here is an attempt to provide this basic understanding and to provide quantitative results that can be used in designing future experiments.

## 1. Introduction

In a recent paper [1] the reaction of slow neutrons with gasous ^3^He was studied experimentally and it was estimated that that in the experiment 46 Lyman alpha photons were produced for each slow neutron (wavelength 0.496 nm) absorbed in a chamber containing ^3^He at a pressure of 93 kPa. It was also noted that the most likely mechanism for the radiation produced was emission produced during the slowing down and charge exchange of the protons and tritons produced in the initial absorption of the neutrons via the reaction

$${}^{3}\text{He}+n\to {}^{3}H^{+}+{}^{1}H^{+}+764\;\text{keV}$$ (1)

and in principle it was possible to calculate the ratios of the various processes which could produce Lyman alpha radiation; i.e., collisions of ^3^H^+^ (tritons), ^1^H^+^ (protons), ^3^H (tritium) and ^1^H (hydrogen atoms) with the background ^3^He gas in the chamber as well as the spatial distribution of the radiation produced. This has now been done and the detailed description of the steps necessary to perform these calculations as well as some results of the calculations will be given here.

The paper is divided into the following parts: The first is a general analysis of the slowing down of the products produced by the neutron absorption described by Eq. (1). The second is concerned with the detailed modelling of the slowing down process specific to the apparatus used in Ref. [1]. The third gives results of calculations and comparison with the experiment of Ref. [1]. The final section will discuss how the modelling may be improved or be used to design other experiments.

## 2. The Slowing Down Model

The reaction of Eq. (1) for a slow neutron produces a proton and a triton moving in opposite directions with the same momentum since practically no momentum is transferred from the neutron. Ignoring the electrons this means that the energies of each proton and triton initially will be 573 keV and 191 keV respectively. Although protons and tritons corresponding to each neutron absorption move in opposite directions, each proton or triton has no particular direction. Furthermore, at these energies the particles will tend to move in straight lines as they lose energy until their energy becomes low enough for elastic collisions to cause them to deviate from straight line paths.

With these considerations in mind, it is useful to investigate a model in which the protons or tritons move in straight lines in a gas (either ^3^He or ^3^He in combination with some other buffer gas) but may change charge as they move along that path and lose energy. Experimentally the charge exchange process has been studied in detail for protons or hydrogen atoms moving in beams for a number of gases [2,3]. For atoms or ions moving in helium gas (^3^He or ^4^He) both the cross sections for charge loss (*σ*_10_) or charge gain (*σ*_01_) have been measured as a function of incident energy. The mean free path for a proton with given energy which picks up a charge is simply Δr = 1/*σ*_01_N where N is the number of atoms per unit volume which is proportional to the pressure. Similarly the mean free path for charge loss for the neutral atom will be the same with *σ*_01_ replaced by *σ*_10_. In addition to the cross sections for charge exchange, the energy loss of a particle per unit path length for protons in helium dE/dr (known as the stopping power) is also known as a function of proton energy [4–6]. Thus the energy loss that occurs in the time between each charge changing collision will be Δr dE/dr. To the extent that the particles move in straight lines the effective range of the particles in slowing down from energy E_0_ to E_f_ is simply the integral of 1/(dE/dr) from E_0_ to E_f_. In our model we assume that protons or tritons initially move a distance given by the mean free path calculated from the pressure and charge transfer cross section as indicated above. During it’s flight in addition to charge transfer it may make a number of elastic or inelastic collisions with the gas which do not result in charge transfer but cause energy loss. The total energy loss can be calculated from the distance travelled and the stopping power. The neutral particle is then assumed to move a distance calculated using the pressure and the charge loss cross section *σ*_01_ and as before, the energy loss for moving that distance is calculated from the stopping power. The two collisions constitute a charge changing cycle resulting in an energy loss which is the sum of the losses in each collision and a change in position which is the sum of the mean free paths. The process is than repeated recording the distance travelled in each charge changing cycle and the energy lost until either the energy of the particle has reached a lower limit or the distance travelled has exceeded a predetermined value. By computing each charge changing collision separately we effectively integrate over the reciprocal of the stopping power by summing over the individual path lengths between collisions and the energy loss for each collision and the total distance travelled corresponds to the effective range. The total number of charge changing cycles which occur during the slowing down process is also obtained. The model described above differs from the way slowing down of tritons and protons following slow neutron absorption via Eq. (1) is usually viewed. The normal procedure [5] is to describe the collisions as heavy particle elastic collisions ignoring the fact that at high energies most of the energy loss is due to the interaction of the fast particle with the atomic electrons of the background gas. This is taken into account by assigning an energy loss to the distance the particle has travelled using the known stopping power. The main object is to determine where the energy loss of the moving particle occurs and the number of electrons produced during the slowing down process. Here we are interested in where during the slowing down process Lyman alpha radiation is produced. We ignore the elastic collisions and hence ignore any deviation from straight line trajectories (straggling), but correctly calculate the energy loss and effective range by using the measured stopping power.

The next step is to estimate the probability of producing Lyman alpha radiation during each charge changing cycle. When the particle is charged, radiation can be produced by direct charge transfer, i.e.,

$$p+\text{He}\to H(2p)+{\text{He}}^{+}\to H(1s)+\text{Lyman}\;\text{alpha}$$ (2)

When the particle is neutral radiation can be produced by direct excitation, i.e.

H+He→H(2p)+He→H(1s)+Lymanalpha (3)

Equations (2) and (3) are written only for protons. We assume that the same processes occur for tritons and tritium atoms and that the cross sections for Lyman alpha production as well as those for charge exchange are the same as those for protons or H atoms at equal energies [7]. The cross sections for all of these processes have been measured as a function of energy and from these cross sections the probability of producing Lyman alpha radiation as neutral or charged particles move a distance Δr can be calculated.

Calculations of the formation of Lyman alpha for protons slowing down in helium is a straightforward procedure, but for the tritons and tritium additional modifications must be made. The stopping power for tritons or tritium used was that for protons moving in helium at one third of the energy. At energies above 10 keV this is reasonable since stopping power has been measured for protons, deuterons and tritons and the results are the same for equal velocities [7]. At energies below 10 keV the data on stopping power may not be reliable [8)].

## 3. The Monte Carlo Model

**Fig. 1:**
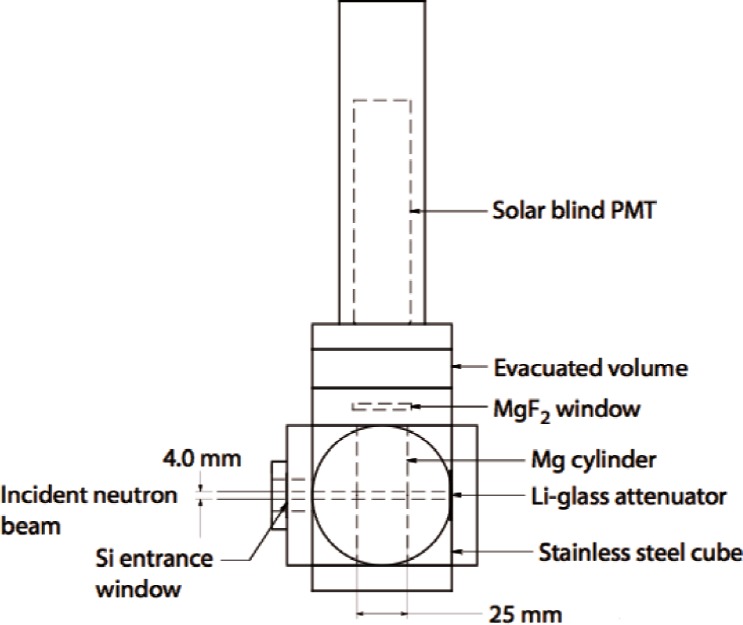
Drawing of the reaction cell used in Ref. [1].

In order to explain how the experiment is modelled it is useful to review the way the results reported in Ref. [1] were obtained. A diagram of the apparatus used in Ref. [1] is reproduced here in Fig. 1. The neutrons enter the 25 mm cylindrical reaction chamber where protons and tritons are produced in a beam of less than 4 mm diameter. Lyman alpha photons are detected which pass through the MgF_2_ window at the top end of the chamber. However, before entering the cylinder the neutrons must first pass through a “dead space” of length Ldead which contains helium at the same pressure and are attenuated there but no protons or tritons produced enter the cylinder and consequently no Lyman alpha radiation produced in the dead space will reach the detector.

The incident neutron flux and its wavelength were measured. From the measured wave length and previous experimental results assuming that the absorption cross section in ^3^He follows a 1/v dependence the cross section at this wavelength is determined. Knowing the neutron cross section and the pressure, the number of neutrons/sec absorbed in the 25 mm path length within the reaction chamber was determined as follows. Let I_0_ be the incident neutron flux. Then

$$I=I_{0}e^{-\text{Ldead}\;\sigma N}(1-e^{-d\sigma N})$$ (4)

will be the flux absorbed in the reaction cell where *σ* is the neutron cross section, N the density of helium atoms in the chamber and Idead and d are the lengths of the dead space and the diameter of the reaction chamber (25 mm) respectively. The first term represents the flux transmitted through the dead space and the second term the flux absorbed within the chamber. Note that I is the absorption produced in the total path length within the reaction chamber.

It was assumed that all photons observed were produced within the reaction chamber and have no preferred direction. If this is true the number of photons observed originating at a particular point will depend on the ratio of the solid angle subtended by the window of the detector at the top of the chamber from that point within the cylinder to 4π. This ratio on average will be the same as that for a point at the center of the cylinder. The solid angle subtended by the detector window for this point is simply 2π(1 − cosΘ) where Θ is the angle whose tangent is the ratio of the window radius (2.7 mm) to the distance of the window from the center of the chamber.

Finally, the attenuation of the window and the efficiency of the detector was accounted for. Measurements were made to estimate these quantities.

We take as a coordinate system *x*, *y* and *z* coordinates with the origin at the center of the 4 mm cylinder describing the neutron path and midway between the walls of the 25 mm cylindrical reaction chamber where radiation is produced with the *z* direction along the axis of the 4 mm cylinder and *x* along the axis of the 25 mm cylinder. *x*_up_ and *x*_down_ are the lengths from the origin to the detector window at the top and to the bottom of the reaction chamber respectively. In our model we assume that there are T point sources of protons or tritons distributed along the *z* axis. The basic idea is to choose the starting points for these point sources randomly and to follow the path of each proton or triton within the chamber as it undergoes charge changing collisions until it either reaches a preset minimum energy or collides with the walls or ends of the reaction chamber. Since the particles are assumed to be emitted isotropically their initial directions are also chosen randomly and they are assumed to move in the same direction until their trajectories are terminated either by falling below a minimum energy or hitting the walls or ends of the 25 mm diameter cylinder containing ^3^He. As they are slowed down within the reaction chamber the probability of emitting Lyman alpha radiation at that point is calculated from the slowing down model and the probability of it’s reaching the detector is estimated.

Two simplifying approximations which can be improved have been made it setting up this model. First, we have ignored the finite dimension of the neutron beam and treated it as a line source. Second, our method of calculating the solid angle for radiation emitted at a point within the chamber is only approximate. There have been a number of detailed calculations of this for various geometries [9,10] and they show that a moderately good approximation to make here is to assume that the effective solid angle for a point source of radiation within the chamber depends only on the distance of the source point from the detector window and we used this approximation to estimate the number of photons entering the detector. A more detailed calculation is possible.

The setting up of the Monte Carlo calculation is straightforward. Depending upon whether protons or tritons are being tracked the incident energy is set to 573 keV or 191 keV and the particle mass to either 1 or 3 u (unified atomic mass unit). Next three random numbers are generated. The first is used to define the starting point of the track on the *z* axis. Since the total path of the neutron beam within the cylinder is d (25 mm) and we have chosen the center of the cylinder as the zero of our coordinate system we choose the initial starting point randomly between − d/2 and d/2. The second and third random numbers define a random direction by choosing a value of cos θ between − 1 and 1 and ϕ between 0 and π. The direction of the particle is then specified after the first charge changing collision by:

x=Δx=Δrsinθcosϕ (5)

y=Δy=Δrsinθsinϕ (6)

z=Δz=Δrcosθ (7)

where Δr is the distance travelled before the first collision and the direction is assumed to remain the same for all subsequent collisions. The path of the particle is then followed as charge changing collisions occur and the energy loss in each collision, the estimated total amount of Lyman alpha radiation formed during the path length Δr, and the fraction of that radiation reaching the detector are calculated and recorded. As in the slowing down model charge changing cycles are then followed until the particle either strikes the walls of the reaction chamber or slows down below a preset minimum energy. The criteria for striking the walls or top or bottom of the cylindrical chamber are simply:

$$z^{2}+y^{2}\geq d^{2}/4$$ (8)

or

$$x\geq x_{\text{up}}$$ (9)

or

$$-x\geq x_{\text{down}}$$ (10)

Once a given track is terminated, either by hitting the walls of the reaction chamber or by the energy falling below the cutoff energy, three more random numbers are generated and a new track begins.

One other correction is made to the procedure described above. Since each track starts at some distance along the *z* axis from its entrance into the 25 mm cylinder (at *z* = − d/2) there are more protons and tritons to be produced at *z* = − d/2 than at *z* = d / 2. We correct for this by computing the flux reduction for a track starting at a given value of *z* and weighting the Lyman alpha production produced by a given track by this reduction.

As will be shown in the next section, typically about 1500 charge changing cycles occur for protons and 2000 for tritons for a cut off energies of 1 keV and 3 keV respectively. For the Monte Carlo calculations 2000 tracks were enough to get a good estimate of the Lyman alpha radiation produced at a given pressure and the calculations typically required about 10 to 20 seconds.

## 4. Results for the Slowing Down Model

The cross sections for charge exchange and for Lyman alpha production via Eq. (2) and Eq. (3). were obtained from the ORNL ‘Redbook’ compilation [11] which is available on line. The cross sections have been fitted to Chebyshev polynomials so that only 11 numbers are needed to obtain a cross section at any energy within the range of the tabulated values. The cross sections for charge exchange were available for the entire energy range from from 600 keV to 1 keV but the cross sections for Lyman alpha production via Eq. (2) and Eq. (3) were only for energies below 90 keV for production by atoms and below 300 keV for production by ions. No attempt was made to estimate Lyman alpha production outside of these ranges.

The stopping power used here for protons in helium came from the NIST compilation of ranges [4] and stopping powers which is also available on line. The tabulation only extends to a lower energy of 1 keV so no results were estimated for final energies below 1 keV for protons and below 3 keV for tritons.

In order to verify the validity of our slowing down model the ranges in helium for protons at 573 keV and tritons at 191 keV were estimated from the NIST compilation for a helium pressure of 101 kPa. The results were 50 mm for ^4^He, 67 mm for ^3^He for protons and 19 mm for tritons in ^3^He.^1^ Calculating directly the distance traveled for protons slowing down to 1 keV and tritons to 3 keV in ^3^He we obtained distances of 67 mm for protons and 18 mm for tritons in good agreement with the compilation values. It is important to note that the effective ranges for ^4^He and ^3^He are different.

**Fig. 2:**
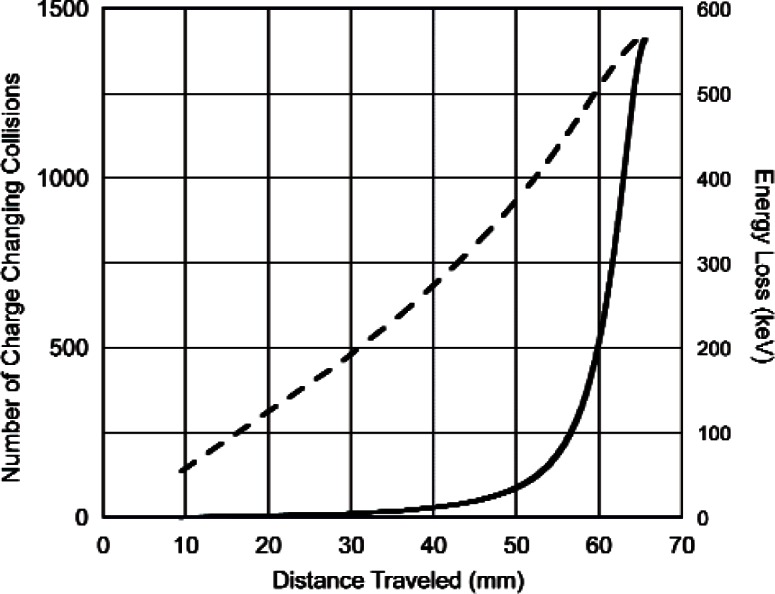
Number of charge changing cycles (_________) and energy loss (__ ___ __) vs. distance (mm) for 573 keV protons slowing down to 1 keV in He^3^ at a pressure of 101 kPa.

**Fig. 3:**
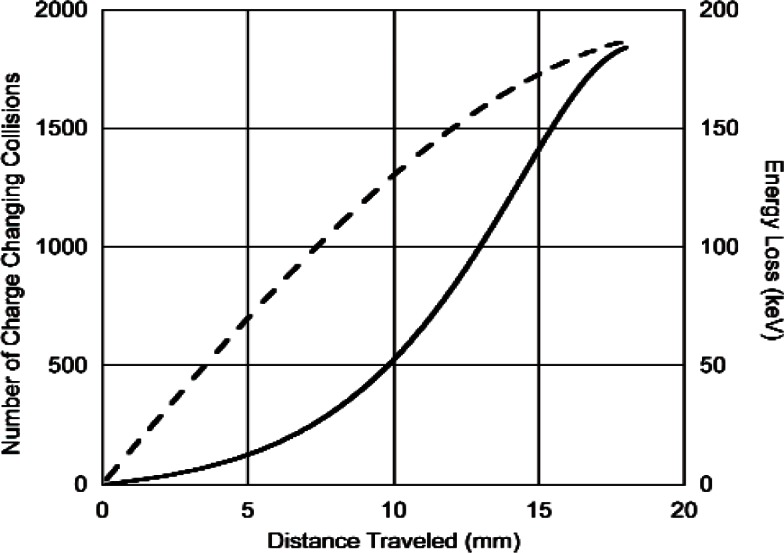
Number of charge changing cycles (_________) and energy loss (__ ___ __) vs. distance (mm) for 191 keV tritons slowing down to 3 keV in He^3^ at a pressure of 101 kPa.

For protons although our model predicts that there will be about 1500 charge changing cycles in slowing down from 573 keV to 1 keV, charge exchange is relatively unimportant as an energy loss mechanism at the higher energies. This is due to the fact that at higher energies the cross section for losing charge is much larger than that for gaining charge [2]. It is only after slowing down to an energy where the two cross sections are equal (~ 100 keV) that energy loss via charge exchange becomes important. This is shown in Fig. 2 where the energy loss for protons in ^3^He at atmospheric pressure (101 kPa) is shown as a function of the distance travelled. The distance travelled before charge exchange is large, i.e., greater than 50 mm at atmospheric pressure. The situation for tritons is completely different as is shown in Fig. 3. There the number of charge changing cycles increases gradually with the energy loss and, as expected, the distance travelled during the slowing down process is much less.

**Fig. 4:**
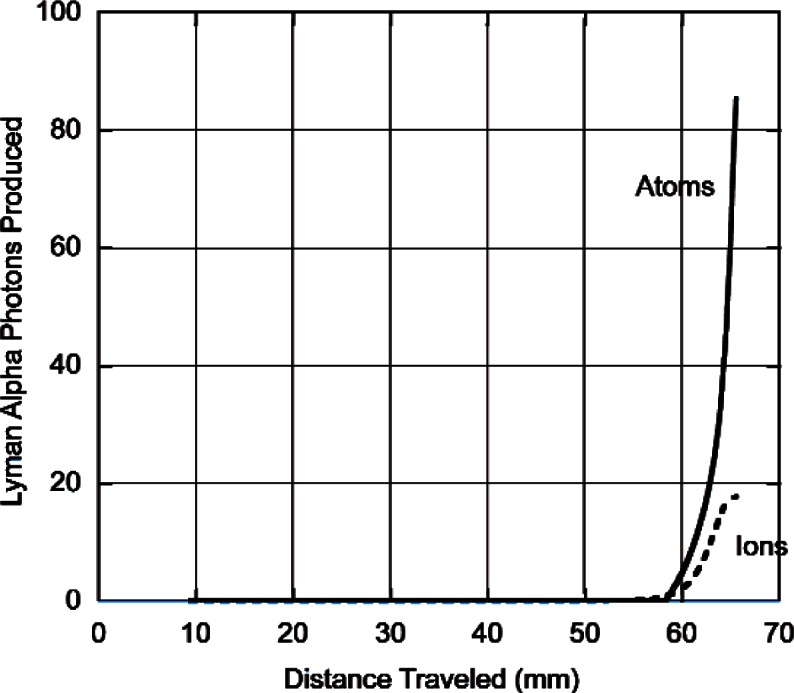
Number of Lyman alpha photons produced vs. distance (mm) by atoms (_________) or ions (__ ___ __) for 573 keV protons slowing down to 1 keV in He^3^ at a pressure of 101 kPa.

**Fig. 5:**
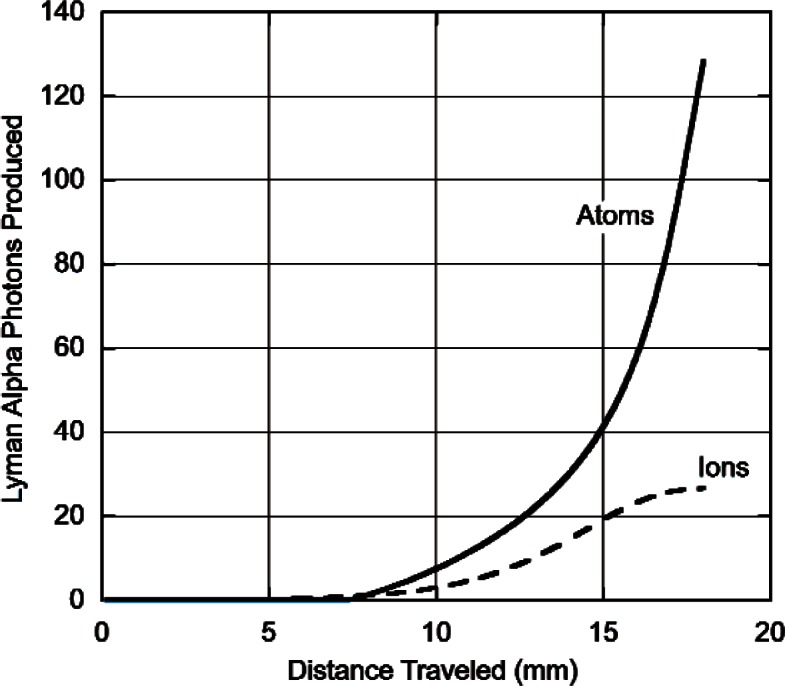
Number of Lyman alpha photons produced vs. distance (mm) by atoms (_________) or ions (__ ___ __) for 191 keV tritons slowing down to 3 keV in He^3^ at a pressure of 101 kPa.

The model predictions for Lyman alpha production (number of photons produced for each neutron absorbed as a function of distance travelled) by atoms or ions is shown for protons and for tritons both at atmospheric pressure in Figs. 4 and 5. What is plotted is the total number of photons produced after the particle has travelled a given distance. The important thing to note here is that for protons, practically no Lyman alpha radiation is produced until the protons have travelled a distance of about 60 mm whereas for tritons there is appreciable production when the particle has travelled a much shorter distance. Within our model, the distance travelled by a particle in losing a given amount of energy will be inversely proportional to the pressure. At lower pressures the particles must travel further before they produce Lyman alpha radiation. In an infinite media the total number of photons produced will be independent of pressure. Nevertheless, the total amount of energy for Lyman alpha production is a small fraction of the total energy loss. These results are summarized in Table 1 which gives the total number of charge changing cycles, the number of Lyman alpha photons produced by neutrals and ions and the percent of the total energy loss due to Lyman alpha production.

## 5. Results for the Monte Carlo Model

There are two ways that we can compare the results of the Monte Carlo model with the experiment described in Ref. [1]. We could, given an incident neutron flux and cross section, use the model to estimate the count rate of photons entering the window of the photon detector. Alternatively we could use the model to predict the total number of photons produced for each absorbed neutron and compare this with the results of Ref. [1]. We have chosen the second alternative since the key results of Ref. [1] are the observations that the reaction yield of Lyman alpha photons per neutron absorption is pressure dependent and much greater than one.

At the pressures used in Ref. [1], the model predicts that there will be no Lyman alpha produced by protons. This is understandable since one can see from Fig. 2 that at atmospheric pressure a proton must travel at least a distance of approximately 55 mm before any Lyman alpha is produced whereas the largest distance that a proton can travel within the reaction chamber is 51 mm. This will also be true at lower pressures, but at higher pressures some signal is to be expected from the protons. We find that at 200 kPa the model predicts for that for 2000 proton trajectories 10 photons would be produced per neutron absorbed even though 1844 of the protons will have hit the walls of the reaction chamber. For slowing down in ^4^He 16 would be produced per neutron absorbed and 1670 would have hit the walls owing to the smaller effective range in ^4^He.

For tritons at atmospheric pressure (101 kPa) the model predicts that 60 photons will be produced for each neutron absorbed which is less than the infinite medium prediction shown in Table 1 of 154. The reason is that for 2000 trajectories 1443 hit the walls of the chamber before they reached the cutoff energy of 3 keV.

**Fig. 6:**
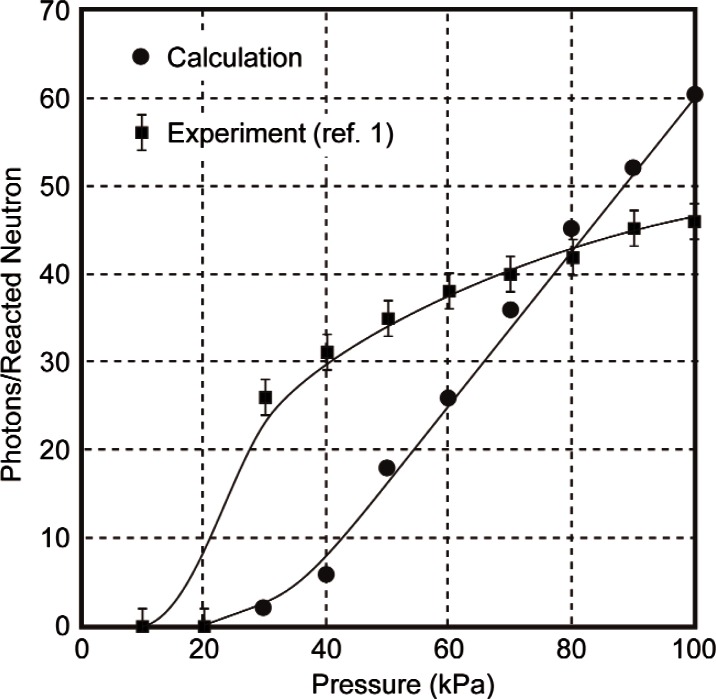
A comparison of the pressure dependence of number of L*α* photons produced per neutron absorbed from the experiment of Ref. [1] and from the model calculation.

The pressure dependence of the model calculation is compared with that of the experiment of Ref. [1] in Table 2 and in Fig. 6. Also shown in the table are the number of triton trajectories that terminate before reaching the cutoff energy of 3 keV. The model results are in moderate agreement with the experiment. At the higher pressures the model predicts approximately the same number of photons/neutrons as the experiment even though over half of the trajectories are terminated. The main difference is that the model predicts a more rapid falloff of the number of photons produced at lower pressures than is observed. Note that the model predicts that all of the tritons will have hit the walls at 40 kPa but that 6 photons/neutron are produced.

At higher pressures one might expect the number of photons observed to approach the infinite medium prediction of 154 photons/neutron since all of the tritons will be slowed down to 3 keV at higher pressure. While this is true, with increasing pressure neutron absorption is much more likely to occur in that part of the chamber nearest the source and thus close to the walls of the cylinder. At high pressure we would expect half of the tritons to hit the wall and the other half to produce photons as if they were moving in an infinite medium. The results shown in Table 2 at the higher pressures show this. The number of photons/neutron produced is roughly one-half of the infinite media results and the number hitting the wall is larger than at lower pressures and approximately one-half of the total number of trajectories.

## 6. Summary and Suggestions for Future Work

The results described in the previous sections are an initial attempt to understand the production of Lyman alpha radiation observed in Ref. [1].

The relatively good agreement of the model calculations at higher pressures with the experimental results is rather gratifying in view of the number of approximations that have been made both in analysing the experiment and in setting up the model calculations. The fact that the calculations underestimate the production at lower pressures seems to indicate that there is another source of Lyman alpha radiation that is not accounted for by the model. One possibility is that no production of Lyman alpha by neutrals was assumed to occur at energies above 90 keV.

Two unanticipated results of the calculations are the prediction that with the present apparatus at pressures below one atmosphere all of the signal is produced by tritons and that different results would be obtained for slowing down in ^4^He due to the change in range. These predictions can be checked using the present apparatus by working at higher pressures and by using a mixture of ^4^He and ^3^He. Experiments using such mixtures are currently being done and will be reported in a future publication.

From the standpoint of designing a cold neutron detector using Lyman alpha radiation, the model described here will be useful. Provided the cross sections and stopping powers for any mixture of gases are known or can be estimated the slowing down model can be used to estimate the total Lyman alpha production and the Monte Carlo model modified to provide predictions of the signal strength at any given detector and neutron source configuration.

## Figures and Tables

**Table 1:** Lyman alpha photons/neutron produced by tritons and protons and the percent of the total energy loss due to Lyman alpha production at a pressure of 101 kPa

Charge changing Cycles		Photons produced by			Energy loss %
		Atoms	Ions	Total	
Tritons	1841	128	26	154	0.082
Protons	1411	85	18	103	0.018

**Table 2:** Comparison of the pressure dependence of the number of Lyman alpha photons produced in the experiment of Ref. [1] and the model calculation. Results are based on 2000 trajectories of tritium at each pressure. The last column gives the number of trajectories which hit the walls before reaching the cutoff energy of 3 keV

Pressure (kPa)	Photons/Neutron (Model)	Photons/Neutron (Ref. [1])	Wall hits
10	0	0	2000
20	0	0	2000
30	2	26	2000
40	6	31	2000
50	18	35	1930
60	26	38	1819
70	36	40	1719
80	45	42	1620
90	52	45	1528
100	60	46	1443
500	78	–	862
660	80	–	842

## Footnotes

1 The ranges given in Ref. [4] are in units of gm/cm^2^. Therefore the range in ^4^He will be 3/4ths the range in ^3^He.
